# 1-(2,6-Diisopropyl­phen­oxy)-4-phenyl­phthalazine

**DOI:** 10.1107/S1600536812033296

**Published:** 2012-07-28

**Authors:** Bihai Tong, Qunying Mei

**Affiliations:** a College of Metallurgy and Resources, Anhui University of Technology, Maanshan 243002, People’s Republic of China; b School of Chemistry and Chemical Engineering, School Library, Anhui University of Technology, Maanshan 243002, People’s Republic of China

## Abstract

In the title mol­ecule, C_26_H_26_N_2_O, the phenyl and phen­oxy rings form dihedral angles of 54.66 (7) and 84.83 (6)°, respectively, with the phthalazine mean plane. The crystal packing exhibits weak C—H⋯π inter­actions.

## Related literature

For details of the synthesis, see: Tong *et al.* (2008 ▶, 2012 ▶). For related structures, see: Dilek *et al.* (2004 ▶); Rajnikant *et al.* (2006 ▶); Sakthivel *et al.* (2011 ▶).
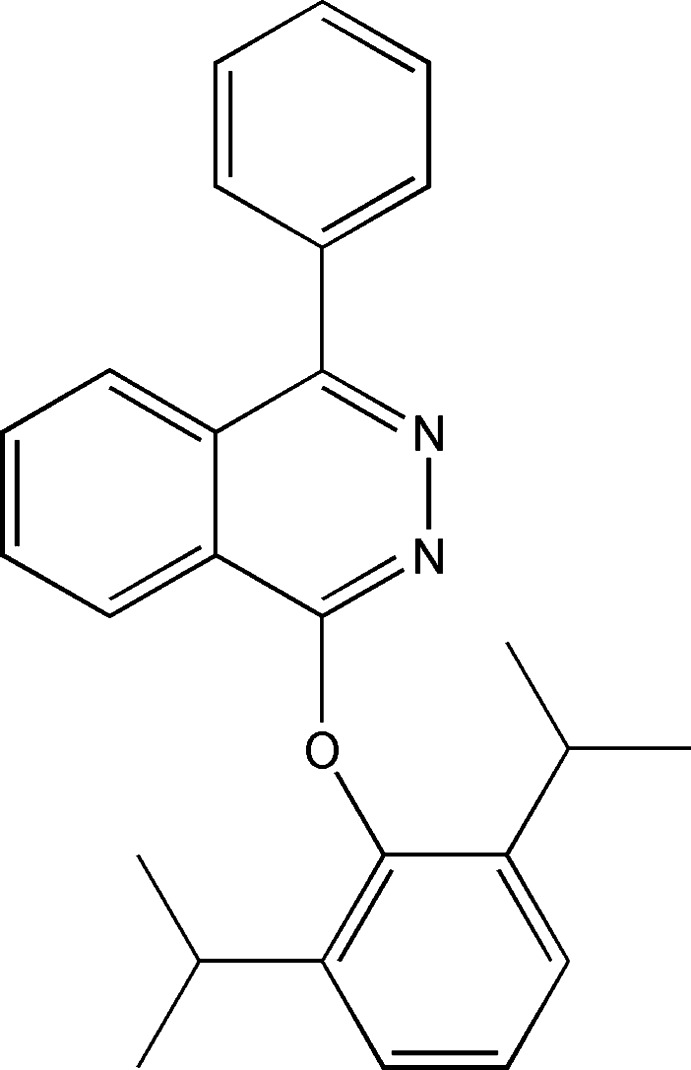


## Experimental

### 

#### Crystal data

C_26_H_26_N_2_O
*M* *_r_* = 382.49
Monoclinic, 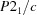

*a* = 14.079 (10) Å
*b* = 8.369 (6) Å
*c* = 19.244 (13) Å
β = 109.104 (9)°
*V* = 2143 (3) Å^3^
*Z* = 4
Mo *K*α radiation
μ = 0.07 mm^−1^
*T* = 273 K
0.31 × 0.29 × 0.14 mm

#### Data collection

Bruker SMART CCD area-detector diffractometer
Absorption correction: multi-scan (*SADABS*; Bruker, 2004 ▶) *T*_min_ = 0.978, *T*_max_ = 0.990
9404 measured reflections
3596 independent reflections
2028 reflections with *I* > 2σ(*I*)
*R*_int_ = 0.036

#### Refinement

*R*[*F* ^2^ > 2σ(*F* ^2^)] = 0.046
*wR*(*F* ^2^) = 0.165
*S* = 0.90
3596 reflections
267 parameters
H-atom parameters constrained
Δρ_max_ = 0.16 e Å^−3^
Δρ_min_ = −0.15 e Å^−3^

### 

Data collection: *SMART* (Bruker, 2004 ▶); cell refinement: *SAINT* (Bruker, 2004 ▶); data reduction: *SAINT*; program(s) used to solve structure: *SHELXS97* (Sheldrick, 2008 ▶); program(s) used to refine structure: *SHELXL97* (Sheldrick, 2008 ▶); molecular graphics: *SHELXTL* (Sheldrick, 2008 ▶); software used to prepare material for publication: *SHELXL97*.

## Supplementary Material

Crystal structure: contains datablock(s) I, global. DOI: 10.1107/S1600536812033296/cv5322sup1.cif

Structure factors: contains datablock(s) I. DOI: 10.1107/S1600536812033296/cv5322Isup2.hkl

Supplementary material file. DOI: 10.1107/S1600536812033296/cv5322Isup3.cml

Additional supplementary materials:  crystallographic information; 3D view; checkCIF report

## Figures and Tables

**Table 1:** Hydrogen-bond geometry (Å, °) *Cg* is the centroid of the C15–C20 ring.

*D*—H⋯*A*	*D*—H	H⋯*A*	*D*⋯*A*	*D*—H⋯*A*
C9—H9⋯*Cg*	0.93	2.77	3.624 (2)	154

**Fig. 1.**
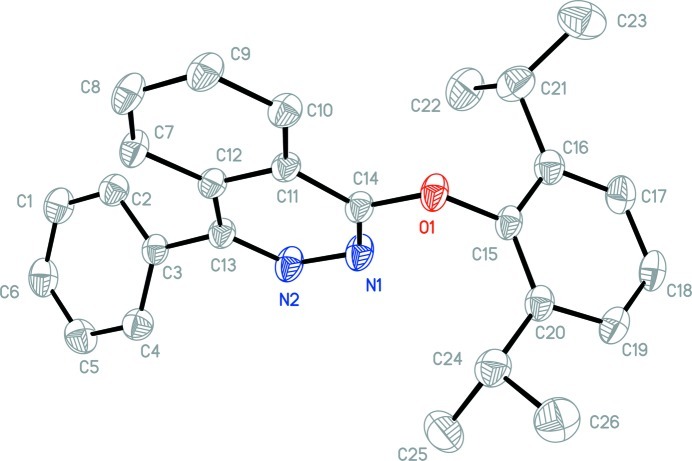
The molecular structure of (I), showing the atomic labeling and 30% probability displacement ellipsoids. H atoms omitted for clarity.

## supplementary crystallographic information

### Comment

Phthalazine is a well known heterocyclic system which is widely used in coordination chemistry and pharmaceutical chemistry. Recently, we have reported the direct synthesis of a series of highly efficient tris-cyclometalated iridium(III) complexes using phenylphthalazine derivatives as ligands (Tong *et al.*, 2008). However, the 2, 6-dimethylphenoxyl groups of phenylphthalazine derivatives hydrolyzate easily in the coordination procedure (Tong *et al.*, 2012). In order to suppress the hydrolyzation process, the title molecule was synthesized as the ligand of cyclometalated iridium(III) complexes.

In the title molecule (Fig. 1), the phthalazine moiety consists of a benzene and a pyridazine rings fused together and shows a planar conformation; the dihedral angle between these rings is 2.00 (6)°. A phenyl and a phenoxyl rings are substituted on the pyridazine ring and dihedral angle of these rings with the pyridazine ring are 54.66 (7) and 84.83 (6)°, respectively. The molecular dimensions in the title compound are in agreement with the corresponding molecular dimensions reported for closely related compounds (Dilek *et al.*, 2004; Rajnikant *et al.*, 2006; Sakthivel *et al.*, 2011). In the crystal, the molecules are held together *via* the weak C—H···π interactions (Table 1).

### Experimental

The title compound was obtained in 89% yield by refluxing 1-chloro-4- phenylphthalazine (4.8 g, 20 mmol), 2,6-diisopropylphenol (2.5 g, 20 mmol) and potassium carbonate (2.8 g, 20 mmol) in *N*,*N*-dimethylformamide (50 ml) at 383 K for 5 h under nitrogen atmosphere. The crystals suitable for crystallographic study were grow from ethanol by slow evaporation at room temperature.

### Refinement

H atoms were positioned geometrically and refined using a riding model, with C—H = 0.95–0.99 Å and with *U*_iso_(H) = 1.2 (1.5 for methyl groups) *U*_eq_(C).

### Crystal data

**Table:**

C_26_H_26_N_2_O	*F*(000) = 816
*M**_r_* = 382.49	*D*_x_ = 1.186 Mg m^−^^3^
Monoclinic, *P*2_1_/*c*	Mo *K*α radiation, λ = 0.71073 Å
Hall symbol: -P 2ybc	Cell parameters from 1750 reflections
*a* = 14.079 (10) Å	θ = 2.7–22.8°
*b* = 8.369 (6) Å	µ = 0.07 mm^−^^1^
*c* = 19.244 (13) Å	*T* = 273 K
β = 109.104 (9)°	Block, colourless
*V* = 2143 (3) Å^3^	0.31 × 0.29 × 0.14 mm
*Z* = 4	

### Data collection

**Table:**

Bruker SMART CCD area-detector diffractometer	3596 independent reflections
Radiation source: fine-focus sealed tube	2028 reflections with *I* > 2σ(*I*)
Graphite monochromator	*R*_int_ = 0.036
ω scans	θ_max_ = 25.0°, θ_min_ = 2.7°
Absorption correction: multi-scan (*SADABS*; Bruker, 2004)	*h* = −16→12
*T*_min_ = 0.978, *T*_max_ = 0.990	*k* = −9→9
9404 measured reflections	*l* = −22→22

### Refinement

**Table:**

Refinement on *F*^2^	Secondary atom site location: difference Fourier map
Least-squares matrix: full	Hydrogen site location: inferred from neighbouring sites
*R*[*F*^2^ > 2σ(*F*^2^)] = 0.046	H-atom parameters constrained
*wR*(*F*^2^) = 0.165	*w* = 1/[σ^2^(*F*_o_^2^) + (0.1*P*)^2^] where *P* = (*F*_o_^2^ + 2*F*_c_^2^)/3
*S* = 0.90	(Δ/σ)_max_ < 0.001
3596 reflections	Δρ_max_ = 0.16 e Å^−^^3^
267 parameters	Δρ_min_ = −0.15 e Å^−^^3^
0 restraints	Extinction correction: *SHELXL97* (Sheldrick, 2008), Fc^*^=kFc[1+0.001xFc^2^λ^3^/sin(2θ)]^-1/4^
Primary atom site location: structure-invariant direct methods	Extinction coefficient: 0.0064 (16)

### Special details

**Table:**

Geometry. All e.s.d.'s (except the e.s.d. in the dihedral angle between two l.s. planes) are estimated using the full covariance matrix. The cell e.s.d.'s are taken into account individually in the estimation of e.s.d.'s in distances, angles and torsion angles; correlations between e.s.d.'s in cell parameters are only used when they are defined by crystal symmetry. An approximate (isotropic) treatment of cell e.s.d.'s is used for estimating e.s.d.'s involving l.s. planes.
Refinement. Refinement of *F*^2^ against ALL reflections. The weighted *R*-factor *wR* and goodness of fit *S* are based on *F*^2^, conventional *R*-factors *R* are based on *F*, with *F* set to zero for negative *F*^2^. The threshold expression of *F*^2^ > σ(*F*^2^) is used only for calculating *R*-factors(gt) *etc*. and is not relevant to the choice of reflections for refinement. *R*-factors based on *F*^2^ are statistically about twice as large as those based on *F*, and *R*- factors based on ALL data will be even larger.

### Fractional atomic coordinates and isotropic or equivalent isotropic displacement parameters (Å^2^)

**Table:**

	*x*	*y*	*z*	*U*_iso_*/*U*_eq_	
O1	0.34098 (10)	0.39831 (14)	0.11904 (7)	0.0538 (4)	
N1	0.21896 (13)	0.28031 (18)	0.02336 (10)	0.0589 (5)	
N2	0.13859 (13)	0.29298 (18)	−0.04103 (10)	0.0586 (5)	
C1	−0.05395 (18)	0.5056 (3)	−0.26486 (13)	0.0669 (7)	
H1	−0.0493	0.5583	−0.3061	0.080*	
C2	0.02474 (16)	0.5119 (2)	−0.19941 (12)	0.0585 (6)	
H2	0.0821	0.5702	−0.1967	0.070*	
C3	0.01925 (14)	0.4318 (2)	−0.13752 (12)	0.0476 (5)	
C4	−0.06648 (16)	0.3467 (3)	−0.14229 (13)	0.0598 (6)	
H4	−0.0711	0.2920	−0.1015	0.072*	
C5	−0.14592 (17)	0.3428 (3)	−0.20810 (15)	0.0703 (7)	
H5	−0.2041	0.2866	−0.2110	0.084*	
C6	−0.13891 (19)	0.4214 (3)	−0.26883 (15)	0.0707 (8)	
H6	−0.1921	0.4175	−0.3129	0.085*	
C7	0.11235 (17)	0.7351 (2)	−0.05706 (12)	0.0573 (6)	
H7	0.0554	0.7465	−0.0982	0.069*	
C8	0.16031 (18)	0.8674 (2)	−0.02080 (13)	0.0628 (7)	
H8	0.1361	0.9684	−0.0379	0.075*	
C9	0.24466 (17)	0.8534 (2)	0.04120 (12)	0.0605 (6)	
H9	0.2776	0.9448	0.0645	0.073*	
C10	0.27973 (15)	0.7063 (2)	0.06820 (12)	0.0519 (6)	
H10	0.3353	0.6973	0.1106	0.062*	
C11	0.23150 (14)	0.5686 (2)	0.03174 (11)	0.0443 (5)	
C12	0.14875 (14)	0.5808 (2)	−0.03242 (11)	0.0452 (5)	
C13	0.10543 (15)	0.4343 (2)	−0.06729 (11)	0.0480 (5)	
C14	0.26126 (14)	0.4091 (2)	0.05578 (11)	0.0471 (5)	
C15	0.38186 (15)	0.2456 (2)	0.14320 (11)	0.0474 (5)	
C16	0.46379 (16)	0.1981 (2)	0.12297 (12)	0.0560 (6)	
C17	0.50782 (17)	0.0537 (3)	0.15145 (14)	0.0668 (7)	
H17	0.5622	0.0166	0.1387	0.080*	
C18	0.47331 (18)	−0.0360 (3)	0.19788 (13)	0.0666 (7)	
H18	0.5039	−0.1328	0.2160	0.080*	
C19	0.39335 (17)	0.0175 (2)	0.21759 (12)	0.0612 (6)	
H19	0.3707	−0.0437	0.2494	0.073*	
C20	0.34555 (15)	0.1613 (2)	0.19098 (11)	0.0517 (6)	
C21	0.5026 (2)	0.2971 (3)	0.07181 (16)	0.0760 (8)	
H21	0.4835	0.4084	0.0759	0.091*	
C22	0.4544 (2)	0.2473 (4)	−0.00709 (16)	0.1097 (11)	
H22A	0.4731	0.1392	−0.0132	0.165*	
H22B	0.4767	0.3166	−0.0384	0.165*	
H22C	0.3826	0.2543	−0.0200	0.165*	
C23	0.6165 (2)	0.2921 (4)	0.09240 (17)	0.1019 (10)	
H23A	0.6368	0.1891	0.0804	0.153*	
H23B	0.6465	0.3109	0.1442	0.153*	
H23C	0.6382	0.3731	0.0656	0.153*	
C24	0.25826 (17)	0.2197 (3)	0.21399 (14)	0.0654 (7)	
H24	0.2486	0.3334	0.2016	0.079*	
C25	0.16130 (18)	0.1335 (4)	0.17186 (16)	0.0937 (9)	
H25A	0.1694	0.0209	0.1815	0.141*	
H25B	0.1459	0.1524	0.1201	0.141*	
H25C	0.1075	0.1729	0.1874	0.141*	
C26	0.2778 (2)	0.2039 (3)	0.29605 (15)	0.0852 (8)	
H26A	0.2816	0.0929	0.3092	0.128*	
H26B	0.2240	0.2535	0.3085	0.128*	
H26C	0.3401	0.2555	0.3224	0.128*	

### Atomic displacement parameters (Å^2^)

**Table:**

	*U*^11^	*U*^22^	*U*^33^	*U*^12^	*U*^13^	*U*^23^
O1	0.0598 (9)	0.0348 (7)	0.0532 (9)	0.0037 (6)	0.0002 (7)	0.0011 (6)
N1	0.0646 (11)	0.0363 (9)	0.0595 (12)	−0.0007 (8)	−0.0018 (9)	0.0003 (8)
N2	0.0653 (11)	0.0372 (9)	0.0587 (12)	−0.0017 (8)	0.0003 (9)	0.0007 (8)
C1	0.0774 (16)	0.0641 (14)	0.0521 (15)	0.0064 (13)	0.0114 (12)	−0.0010 (11)
C2	0.0643 (13)	0.0506 (12)	0.0563 (15)	−0.0018 (10)	0.0138 (11)	−0.0013 (11)
C3	0.0519 (12)	0.0370 (10)	0.0505 (14)	0.0041 (9)	0.0121 (10)	−0.0036 (9)
C4	0.0614 (14)	0.0543 (13)	0.0605 (15)	−0.0038 (11)	0.0155 (12)	−0.0015 (11)
C5	0.0563 (14)	0.0654 (15)	0.0784 (19)	−0.0089 (12)	0.0072 (13)	−0.0137 (13)
C6	0.0710 (16)	0.0656 (15)	0.0588 (17)	0.0075 (13)	−0.0016 (13)	−0.0094 (12)
C7	0.0664 (13)	0.0404 (11)	0.0563 (14)	0.0078 (10)	0.0081 (11)	0.0039 (10)
C8	0.0878 (16)	0.0348 (10)	0.0587 (15)	0.0072 (11)	0.0145 (13)	0.0019 (10)
C9	0.0790 (16)	0.0352 (11)	0.0620 (15)	−0.0052 (10)	0.0157 (13)	−0.0046 (10)
C10	0.0599 (13)	0.0419 (11)	0.0499 (13)	−0.0044 (9)	0.0127 (10)	−0.0039 (9)
C11	0.0489 (11)	0.0346 (10)	0.0489 (13)	−0.0002 (8)	0.0150 (10)	0.0007 (8)
C12	0.0499 (11)	0.0380 (10)	0.0471 (13)	0.0017 (9)	0.0151 (10)	0.0000 (8)
C13	0.0511 (11)	0.0382 (10)	0.0516 (14)	0.0016 (9)	0.0124 (10)	0.0012 (9)
C14	0.0503 (12)	0.0363 (10)	0.0499 (13)	0.0009 (9)	0.0100 (10)	0.0011 (9)
C15	0.0531 (12)	0.0356 (10)	0.0431 (12)	0.0004 (9)	0.0015 (10)	−0.0001 (8)
C16	0.0579 (13)	0.0504 (12)	0.0547 (14)	0.0041 (11)	0.0115 (11)	−0.0023 (10)
C17	0.0661 (14)	0.0667 (14)	0.0645 (16)	0.0174 (12)	0.0169 (12)	0.0064 (12)
C18	0.0709 (15)	0.0567 (13)	0.0616 (16)	0.0210 (12)	0.0072 (13)	0.0125 (11)
C19	0.0735 (15)	0.0501 (12)	0.0541 (15)	0.0063 (11)	0.0128 (12)	0.0103 (10)
C20	0.0558 (12)	0.0428 (11)	0.0486 (13)	0.0022 (9)	0.0062 (10)	−0.0012 (9)
C21	0.0927 (19)	0.0602 (14)	0.086 (2)	0.0058 (13)	0.0440 (16)	0.0081 (13)
C22	0.117 (2)	0.139 (3)	0.069 (2)	0.004 (2)	0.0252 (18)	0.0288 (19)
C23	0.095 (2)	0.127 (3)	0.095 (2)	−0.0228 (19)	0.0463 (18)	−0.0186 (19)
C24	0.0721 (15)	0.0506 (12)	0.0747 (18)	0.0056 (11)	0.0255 (13)	0.0055 (11)
C25	0.0593 (16)	0.127 (2)	0.092 (2)	0.0058 (16)	0.0200 (15)	0.0062 (18)
C26	0.0941 (19)	0.0898 (19)	0.074 (2)	−0.0024 (15)	0.0309 (16)	−0.0057 (15)

### Geometric parameters (Å, º)

**Table:**

O1—C14	1.362 (2)	C15—C20	1.383 (3)
O1—C15	1.416 (2)	C15—C16	1.391 (3)
N1—C14	1.289 (2)	C16—C17	1.386 (3)
N1—N2	1.382 (2)	C16—C21	1.519 (3)
N2—C13	1.311 (2)	C17—C18	1.372 (3)
C1—C6	1.369 (3)	C17—H17	0.9300
C1—C2	1.380 (3)	C18—C19	1.375 (3)
C1—H1	0.9300	C18—H18	0.9300
C2—C3	1.390 (3)	C19—C20	1.392 (3)
C2—H2	0.9300	C19—H19	0.9300
C3—C4	1.379 (3)	C20—C24	1.517 (3)
C3—C13	1.492 (3)	C21—C22	1.505 (4)
C4—C5	1.389 (3)	C21—C23	1.522 (4)
C4—H4	0.9300	C21—H21	0.9800
C5—C6	1.373 (4)	C22—H22A	0.9600
C5—H5	0.9300	C22—H22B	0.9600
C6—H6	0.9300	C22—H22C	0.9600
C7—C8	1.364 (3)	C23—H23A	0.9600
C7—C12	1.412 (3)	C23—H23B	0.9600
C7—H7	0.9300	C23—H23C	0.9600
C8—C9	1.386 (3)	C24—C26	1.518 (4)
C8—H8	0.9300	C24—C25	1.522 (3)
C9—C10	1.364 (3)	C24—H24	0.9800
C9—H9	0.9300	C25—H25A	0.9600
C10—C11	1.403 (3)	C25—H25B	0.9600
C10—H10	0.9300	C25—H25C	0.9600
C11—C12	1.397 (3)	C26—H26A	0.9600
C11—C14	1.430 (3)	C26—H26B	0.9600
C12—C13	1.435 (3)	C26—H26C	0.9600
			
C14—O1—C15	118.74 (13)	C15—C16—C21	122.17 (19)
C14—N1—N2	118.87 (15)	C18—C17—C16	121.8 (2)
C13—N2—N1	119.92 (15)	C18—C17—H17	119.1
C6—C1—C2	119.8 (2)	C16—C17—H17	119.1
C6—C1—H1	120.1	C17—C18—C19	119.9 (2)
C2—C1—H1	120.1	C17—C18—H18	120.1
C1—C2—C3	120.7 (2)	C19—C18—H18	120.1
C1—C2—H2	119.6	C18—C19—C20	121.4 (2)
C3—C2—H2	119.6	C18—C19—H19	119.3
C4—C3—C2	119.00 (19)	C20—C19—H19	119.3
C4—C3—C13	120.14 (19)	C15—C20—C19	116.5 (2)
C2—C3—C13	120.84 (19)	C15—C20—C24	122.73 (18)
C3—C4—C5	119.9 (2)	C19—C20—C24	120.8 (2)
C3—C4—H4	120.0	C22—C21—C16	111.3 (2)
C5—C4—H4	120.0	C22—C21—C23	110.1 (2)
C6—C5—C4	120.3 (2)	C16—C21—C23	112.9 (2)
C6—C5—H5	119.9	C22—C21—H21	107.4
C4—C5—H5	119.9	C16—C21—H21	107.4
C1—C6—C5	120.3 (2)	C23—C21—H21	107.4
C1—C6—H6	119.9	C21—C22—H22A	109.5
C5—C6—H6	119.9	C21—C22—H22B	109.5
C8—C7—C12	120.4 (2)	H22A—C22—H22B	109.5
C8—C7—H7	119.8	C21—C22—H22C	109.5
C12—C7—H7	119.8	H22A—C22—H22C	109.5
C7—C8—C9	120.84 (19)	H22B—C22—H22C	109.5
C7—C8—H8	119.6	C21—C23—H23A	109.5
C9—C8—H8	119.6	C21—C23—H23B	109.5
C10—C9—C8	120.39 (18)	H23A—C23—H23B	109.5
C10—C9—H9	119.8	C21—C23—H23C	109.5
C8—C9—H9	119.8	H23A—C23—H23C	109.5
C9—C10—C11	119.66 (19)	H23B—C23—H23C	109.5
C9—C10—H10	120.2	C20—C24—C26	112.8 (2)
C11—C10—H10	120.2	C20—C24—C25	111.3 (2)
C12—C11—C10	120.60 (16)	C26—C24—C25	109.8 (2)
C12—C11—C14	115.19 (16)	C20—C24—H24	107.6
C10—C11—C14	124.21 (18)	C26—C24—H24	107.6
C11—C12—C7	118.01 (16)	C25—C24—H24	107.6
C11—C12—C13	117.06 (16)	C24—C25—H25A	109.5
C7—C12—C13	124.90 (18)	C24—C25—H25B	109.5
N2—C13—C12	123.21 (18)	H25A—C25—H25B	109.5
N2—C13—C3	114.73 (16)	C24—C25—H25C	109.5
C12—C13—C3	122.06 (16)	H25A—C25—H25C	109.5
N1—C14—O1	119.47 (16)	H25B—C25—H25C	109.5
N1—C14—C11	125.72 (18)	C24—C26—H26A	109.5
O1—C14—C11	114.81 (16)	C24—C26—H26B	109.5
C20—C15—C16	124.11 (18)	H26A—C26—H26B	109.5
C20—C15—O1	118.68 (18)	C24—C26—H26C	109.5
C16—C15—O1	116.85 (18)	H26A—C26—H26C	109.5
C17—C16—C15	116.3 (2)	H26B—C26—H26C	109.5
C17—C16—C21	121.5 (2)		

### Hydrogen-bond geometry (Å, º)

Cg is the centroid of the C15–C20 ring.

**Table:**

*D*—H···*A*	*D*—H	H···*A*	*D*···*A*	*D*—H···*A*
C9—H9···*Cg*	0.93	2.77	3.624 (2)	154
